# Predictive Factors of Prolonged Ventilation Following Cardiac Surgery with Cardiopulmonary Bypass

**DOI:** 10.21470/1678-9741-2020-0164

**Published:** 2021

**Authors:** Rezan Aksoy, Ayse Zehra Karakoc, Deniz Cevirme, Ahmet Elibol, Fatih Yigit, Üzeyir Yilmaz, Murat Bulent Rabus

**Affiliations:** 1 Department of Cardiovascular Surgery, University of Health Sciences, Kosuyolu Heart Education and Research Hospital, Istanbul, Turkey.

**Keywords:** Cardiopulmonary Bypass, Respiration, Artificial, Perfusion, Intubation, Intratracheal, Airway Extubation, Hemorrhage

## Abstract

**Introduction:**

In this trial, we initially aimed to investigate the major predictive factors for prolonged mechanical ventilation (PMV) following cardiac surgery with cardiopulmonary bypass (CPB) in our center and, secondarily, we tried to find out the effects of the independent factors on mortality.

**Methods:**

Between July 2017 and August 2018, 207 patients who underwent cardiac surgery with CPB were retrospectively investigated. The patients were randomly divided into two subgroups according to the duration of ventilator dependence (group 1 <24 hours, n=164, 79%; group 2 >24 hours, n=43, 21%).

**Results:**

207 patients (mean age 59.47±10.56) who underwent cardiac surgery with CPB were enrolled in this study (n=145, 70% of male patients; n=62, 30% of female patients). Amid these patients, 43 (n=43, 20.77%) had prolonged intubation time. After multivariate logistic regression analysis among preoperative factors, female gender (OR=2.321, *P*=0.028), leukocytosis (OR=1.233, *P*=0.006), perioperative lactate level (OR=1.224, *P*=0.027), CPB time (OR=1.012, *P*=0.012) and postoperative revision for bleeding (OR=23.125, *P*=0.040) were significantly detected. The effect of predictive factors on mortality after cardiac surgery was determined and found that PMV did not affect hospital mortality (OR=1.979, *P*=0.420).

**Conclusion:**

In our report, we revealed, differently from previous studies, that intraoperative lactate levels which manifest organ perfusion and oxygenation were included and were significantly different in the early extubation group compared to the PMV group. Female gender, preoperative leukocytosis, intraoperative CPB time, lactate levels and postoperative revision for bleeding were the independent predictive factors for PMV. Moreover, PMV did not affect the early-term mortality during hospital stay.

**Table:** 

Abbreviations, acronyms & symbols				
**ACX** **ARF** **ANOVA** **BMI** **CABG** **COPD** **CPB** **CRP** **EF**	**= Aortic cross-clamp** **= Acute renal failure** **= Analysis of variance** **= Body mass index** **= Coronary artery bypass grafting** **= Chronic obstructive pulmonary disease** **= Cardiopulmonary bypass** **= C-reactive protein** **= Ejection fraction**		**FEV1** **HT** **ICU** **NAMDRC** **NYHA** **PMV** **PVD**	**= Forced expiratory volume in 1 second** ** = Hypertension** **= Intensive care unit** **= National Association for Medical Direction of** **Respiratory Care** **= New York Heart Association** **= Prolonged mechanical ventilation** **= Peripheral vascular disease**

## INTRODUCTION

Cardiac surgery with cardiopulmonary bypass (CPB) induces an abnormal inflammatory response that culminates in multiple organ dysfunction, especially lung dysfunction. However, there is no consensus about whether CPB itself is totally liable for the impairment in lungs’ functions after open heart surgery ^[1]^. After cardiac surgery, patients are weaned from mechanical ventilator in the intensive care unit (ICU). Ventilator dependence following coronary artery bypass grafting (CABG) is often associated with significant morbidity and mortality ^[2,3]^. Prolonged postoperative ventilation time, commonly accepted as longer than 24 hours after isolated CABG, is a constituent part of the STS (Society of Thoracic Surgeons) CABG composite score and must be regarded as an undesired outcome ^[5,6]^. Meta-analysis showed that there is no common threshold timing for the ideal extubation after cardiac surgery, including every different types of procedures such as valve surgeries besides isolated CABG. Furthermore, according to a worldwide meta-analysis, previous studies accepted very different time thresholds such as 6 hours or 21 days after operation ^[7,8]^. Recently, there were enormous improvements in surgical techniques in cardiac surgery and highly regenerated postoperative management in the ICU; however, ventilator dependence is still a significant problem of the postoperative period. In addition, prolonged mechanical ventilation (PMV) after cardiac surgery is a significant financial burden for the hospital due to the long ICU stay and hospitalization duration. The prevalence of PMV was noted in previous cohort studies between 6.2% and 7.3% ^[6]^. The substantial part of patients with PMV requires tracheostomy, long ICU care and, ultimately, much longer hospital stay and need for recurrent hospitalization ^[9]^.

Postoperative management of ventilator dependence is multifactorial. Characteristics, habits and diseases of patients can affect the duration of mechanical ventilator support after surgery. The difficulty of surgical procedure, total CPB time and other intraoperative conditions can affect the functions of the lungs after cardiac surgery and PMV occurs as a result. In this trial, we initially aimed to investigate the major predictive factors of PMV following cardiac surgery with CPB in our center and, secondarily, we tried to find out the effects of the independent factors on mortality.

## METHODS

### Patient Population and Data

Between July 2017 and August 2018, 207 patients who underwent cardiac surgery with cardiopulmonary bypass were retrospectively investigated. Patient data were searched on our own hospital records and archive systems.

### Prolonged Mechanical Ventilation

The patients were randomly divided into two subgroups according to the duration of ventilator dependence (group 1 <24 hours, n=164, 79%; group 2 >24 hours, n=43, 21%). Patients who underwent complex aortic surgery, congenital heart surgery, pulmonary endarterectomy and surgery for heart failure were excluded. In addition, patients who had ventilator dependence or had undergone tracheostomy before the surgery were also excluded.

Preoperative demographic data were obtained, including smoking (within 4 weeks of surgery), diabetes mellitus (need for oral medication or insulin prior to cardiac surgery), hypertension (HT), chronic obstructive pulmonary disease (COPD), acute renal failure (creatinine >2.5 mg/dl or requiring hemodialysis). The dual bronchodilator therapy (inhaler steroid and beta-agonist) was applied for at least three days before surgery and as a substantial part of respiratory physiotherapy; breathing exercises were performed until discharge. Blood albumin level (mg/dl), blood troponin level, C-reactive protein (CRP), white blood cell count (WBC), hemoglobin level and forced expiratory volume in 1 second (FEV1) in pulmonary function tests were evaluated. Perioperative lactate level, CPB and cross-clamp times (minutes) and blood transfusion count (unit) were registered. Postoperative ventilation time (hours), atrial fibrillation (AF), blood transfusion (unit), presence of infection (surgical/pulmonary disease), need for revision (surgical/bleeding), need for hemodialysis, cerebrovascular events (CVE), length of stay in the hospital and in the intensive care unit (ICU) and mortality were recorded for each group in the short-term postoperative period (one month).

### Anesthetic Protocols

Initially, anesthesia was started with a preoxygenation procedure with 3 deep breaths and subsequently it was induced with midazolam (0.5 mg/kg), fentanyl (5 µg/kg), propofol (1 mg/kg) and rocuronium (0.6 µg/kg) intravenously. Afterward, maintenance of inhalation anesthesia was provided with sevoflurane (minimum alveolar concentration 2%) and, for the continuous neuromuscular blockade, rocuronium (0.2 mg/kg) was applied intermittently for 20 minutes and, if necessary, fentanyl (100 µg/kg) was applied as well.

### Extubation Criteria

The patient must be hemodynamically stabilized, without excessive bleeding (less than 50 ml/h), fully awake, moving the upper and lower limbs easily and without signs of neurological deficit. Arterial blood gas values as pH>7.35, PaCO_2_<40 mmHg and PaO_2_>70 mmHg were required. In addition, the patient needed to have a tidal volume of 6-8 ml/kg and a peak negative inspiratory pressure ≤20 cmH_2_O. Prior to extubation, the chest X-ray was examined carefully to not neglect pneumothorax, atelectasis or severe pleural effusion. The critical care physician in charge of the cardiac surgery ICU was responsible for the final decision to extubate the patient after a successful attempt of spontaneous ventilation and confirmation of normal neurological findings. Similarly, the decision to reintubate the patient was at the discretion of the attending intensivist in the cardiac ICU.

### Surgical Procedure

All surgical procedures were performed with cardiopulmonary bypass during cardiac surgery. In all patients, a traditional median sternotomy was applied. We used CPB in all patients. Moderate systemic hypothermia (28°C-30°C) was used. In CPB, the flow rate was maintained as 2.2-2.5 L/min/m^2^, the mean perfusion pressure was 50-80 mmHg, and the required hematocrit levels were 20%-25%. Myocardial protection was administered with intermittent antegrade and continuous retrograde technique via hypothermic and hyperkalemic blood cardioplegia.

### Statistical Analysis

For statistical analysis, the NCSS 2007 software (Number Cruncher Statistical System, Kaysville, Utah, USA) was used. When evaluating the study data, descriptive statistical methods (mean, standard deviation, median, frequency, percentage, minimum, maximum) were used; for quantitative data, Student’s t-test was used to compare the 2 groups of variables with normal distribution, and the Mann-Whitney U test was used for variables without a normal distribution. In the comparison of 3 or more groups without normal distribution, the Kruskal-Wallis test was applied, followed by the Mann-Whitney U test to determine which group the difference originated from. In the comparison of qualitative data, Pearson’s chi-square test, Fisher-Freeman-Halton test, Fisher’s exact test and Yates’ correction for continuity test (Yates’ corrected chi-square) were applied. For independent group comparisons, one-way analysis of variance (ANOVA) was used for parametric continuous data, while the Kruskal-Wallis test was used for non-parametric continuous data and the Pearson chi-square test was used for categorical data. ‘Post hoc tests’ were utilized for checking the analyses. To determine independent predictors for dependent variables, subsequently to univariate analysis test results, logistic regression analysis was applied to determine the ultimate risk factors and odds ratios of the factors foreseeing PMV after cardiac surgery.

## RESULTS

Two hundred and seven patients (mean age 59.47±10.56) who underwent cardiac surgery with CPB were enrolled in this study (n=145, 70% of male patients; n=62, 30% of female patients). Amid these patients, 43 (n=43, 20.77%) had prolonged intubation time. In this study protocol, PMV was described as extubation time after the first 24 hours after cardiac surgery due to the previous literature. All distributions of demographic data with univariate analysis and preoperative, perioperative and postoperative characteristics are shown in Tables 1, 2 and 3.

**Table 1:** Preoperative factors in PMV and early extubation group.

		Early extubation group <24h	PMV group >24h	Total	*P*
		(n=164)	(n=43)		
**Gender**	Female	43 (69.4%)	19 (30.6%)	62 (100%)	**0.022**
	Male	121 (83.4%)	24 (16.6%)	145 (100%)	
Smoking	Present	69 (85%)	12 (15%)	81 (100%)	0.126
	Absent	95 (76.2%)	31 (23.8%)	126 (100%)	
Diabetes mellitus	Present	74 (81.3%)	17 (18.7%)	91 (43.3%)	0.511
	Absent	90 (77.6%)	26 (22.4%)	116 (100%)	
Hypertension	Present	83 (83%)	17 (17%)	100 (100%)	0.196
	Absent	81 (75.7%)	26 (24.3%)	107 (100%)	
COPD	Present	20 (90.9%)	2 (9.1%)	22 (10.5%)	0.153
	Absent	144 (77.8%)	41 (22.2%)	185 (100%)	
Stroke	Present	8 (66.7%)	4 (33.3%)	12 (100%)	0.270
	Absent	156 (80%)	39 (20%)	195 (100%)	
**Emergency surgery**	Present	3 (30%)	7 (70.07%)	10 (100%)	**<0.001**
	Absent	159 (81.5%)	36 (18.5%)	195 (100%)	
Age		59.2±10.6	60.8±10.2	59.5±10.5	0.252
BMI		28.5±4.2	27.7±4.6	28.3±4.3	0.195
Blood glucose level		142.6±66.1	150.5±77.6	144.3±68.5	0.886
Preop. creatinine		9.5±103	1.3±1.4	7.8±91.7	0.278
Hgb		12.9±2.1	12.4±2.3	12.8±2.2	0.246
**Leukocytosis**		8.5±2.4	10.2±3.1	8.8±2.7	0.001
Platelet		238.7±71.6	251.5±90.4	241.4±75.9	0.735
Lymphocytes		2±0.8	2±0.9	2±0.8	0.85
Monocytes		0.7±0.6	0.7±0.3	0.7±0.5	0.316
Lymphocyte/monocyte ratio		3.5±1.8	3.3±1.8	3.4±1.8	0.49
**Albumin**		4±0.6	3.8±0.5	4±0.6	**0.042**
Preop troponin		0.5±2	1.1±3.6	0.6±2.4	0.095
CRP		3±4.2	3.4±4.1	3.1±4.2	0.55
EF (%)		56±10.8	55.5±11.1	55.9±10.8	0.592
FEV1		89.6±14.9	86.4±14.5	89±14.8	0.27

BMI=body mass index; COPD=chronic obstructive pulmonary disease; CRP=C-reactive protein; EF=ejection fraction; FEV1=forced expiratory volume in 1 second; Hgb=hemoglobin; PMV=prolonged mechanical ventilation Chi-square test, Mann-Whitney U test and Student's t-test were performed and a *P* <0.05 was considered significant.

**Table 2:** Perioperative factors in PMV and early extubation groups.

	Early extubation <24h	PMV >24h	Total	*P*
**Lactate**	3.1±1.8	4.6±2.6	3.4±2.1	**<0.001**
**CPB time (min.)**	126.9±49.3	167.5±62.3	135.1±54.6	**<0.001**
**ACX time (min.)**	83.7±75.3	98.3±55.4	86.7±71.7	0.030
**Periop. mean pressure**	87.5±64.9	79.7±11.8	85.9±58.1	0.047

Mann-Whitney U test and Student's t-tests were performed and a *P* <0.001 was considered significant. ACX=aortic cross-clamp; CPB=cardiopulmonary bypass; PMV=prolonged mechanical ventilation

**Table 3:** Postoperative factors in PMV and early extubation groups.

		Early extubation <24h	PMV >24h	Total	*P*
Atrial fibrillation	Present	%	8 (29.6%)	27 (100%)	0.224
	Absent	145 (80.6%)	35 (19.4%)	180 (100%)	
Acute renal failure	Present	16 (50%)	16 (50%)	32 (100%)	**<0.001**
	Absent	148 (84.6%)	27 (15.4%)	175 (100%)	
Lung infection	Present	6 (31.6%)	13 (68.4%)	19 (100%)	**<0.001**
	Absent	158 (84.0%)	30 (16.0%)	188 (100%)	
Surgical infection	Present	11 (64.7%)	5 (31.3%)	16 (100%)	0.283
	Absent	153 (80.1%)	38 (19.9%)	191 (100%)	
**Revision for bleeding**	Present	5 (35.7%)	9 (64.3%)	14 (100%)	**<0.001**
	Absent	159 (82.4%)	34 (17.6%)	193 (100%)	
**Revision for bleeding or revision of surgical area**	Present	3 (27.3%)	8 (72.7%)	11 (100%)	**<0.001**
	Absent	161 (82.1%)	35 (17.9%)	196 (100%)	
**Blood culture positivity**	Present	12 (44.4%)	15 (55,6%)	27 (100%)	**<0.001**
	Absent	152 (84.4%)	28 (15.6%)	180 (100%)	
**Postop. creatinine**		1.5±2.4	1.5±1.2	1.5±2.2	**0.005**
Postop. leukocytosis		12.5±7.7	14.1±5.2	12.8±7.3	0.588
**Postop. troponin**		24.6±258	46.1±224.5	29.1±251.1	**0.032**
Postop. CRP		5.5±5.2	6.3±5.4	5.7±5.3	0.587
Postop. EF (%)		55.9±8.8	54.6±9.0	55.7±88.1	0.453
**Length of ICU stay**		5.2±6.7	16.5±15.6	7.6±10.3	<0.001
**Length of hospital stay**		13±9.8	23.9±16	15.3±12.2	<0.001
Blood infusion (units)		1.4±1.2	2.2±2.5	1.5±1.6	0.067
Mortality	Present	9 (34.6%)	16 (64%)	26 (12.4%)	**<0.001**
	Absent	155 (85.2%)	27 (14.8%)	182 (100%)	

CRP=C-reactive protein; EF=ejection fraction; ICU=intensive care unit; PMV=prolonged mechanical ventilation

In our preoperative study model, gender, smoking, diabetes mellitus, HT, COPD, stroke, emergency, age, BMI, blood glucose level, preoperative albumin, troponin, CRP, ejection fraction (EF) and FEV1 values were included and evaluated. Perioperative CPB time, intraoperative lactate levels, cross-clamp time and mean arterial pressure were included. Postoperative acute renal failure (ARF), lung infection, surgical infection, acute renal failure, revision for bleeding, revision of the surgical area, blood culture positivity, postoperative creatinine, troponin, CRP, EF, leukocytosis and blood transfusion units, length of ICU stay and total hospitalization time were implicated in this study. In addition, one-way ANOVA test was used to compare types of operation with extubation times. As the distribution among the groups was homogeneous (Levene’s test *P*>0.05), the relationships among the groups were applied with Tukey test. Accordingly, there was no significant difference among the types of operation in terms of extubation times (*P*>0.05).

Among the preoperative factors, female gender, emergency surgery, albumin levels and leukocytosis; intraoperative CPB time and lactate levels; postoperative lung infection, acute renal failure, blood culture positivity, revision for bleeding or revision of the surgical area, postoperative creatinine levels and troponin levels were significantly different between PMV and early extubation groups (*P*<0.001).

After multivariate logistic regression analysis among preoperative factors, female gender (OR=2.321, *P*=0.028), leukocytosis (OR=1.233, *P*=0.006), perioperative lactate level (OR=1.224, *P*=0.027), CPB time (OR=1.012, *P*=0.012) and postoperative revision for bleeding (OR=23.125, *P*=0.040) were detected significantly and shown in Tables 4, 5 and 6.

**Table 4:** Logistic regression analysis - preoperative factors.

	OR	95% CI		*P*
		Lower	Upper	
*Female gender*	*2.321*	*1.093*	*4.927*	*0.028*
*Leukocytosis*	*1.233*	*1.060*	*1.433*	*0.006*
Albumin	0.630	0.328	1.210	0.165
Emergency surgery	0.319	0.060	1.692	0.180

**Table 5:** Logistic regression analysis - perioperative factors.

	OR	95% CI		*P*
		Lower	Upper	
*Lactate*	*1.224*	*1.023*	*1.465*	*0.027*
*CPB*	*1.012*	*1.003*	*1.022*	*0.012*
ACX	0.996	0.986	1.006	0.405
Periop. mean pressure	0.976	0.944	1.009	0.150

ACX=aortic cross-clamp; CPB=cardiopulmonary bypass

**Table 6:** Logistic regression analysis - postoperative factors.

	OR	95% CI		*P*
		Lower	Upper	
Acute renal failure	1.063	0.107	10.524	0.958
Lung infection	1.673	0.062	45.229	0.760
*Revision for bleeding*	23.125	1.159	461.258	*0.040*
Revision for bleeding or revision of surgical area	10.777	0.291	399.428	0.197
Blood culture positivity	0.720	0.053	9.742	0.805
Postop. creatinine	0.745	0.438	1.264	0.275
Postop. troponin	1.000	0.998	1.001	0.744
Length of ICU stay	1.079	0.958	1.215	0.210
Length of hospital stay	1.038	0.937	1.149	0.475
ICU=intensive care unit				

The effect of the predictive factors on mortality after cardiac surgery was determined and it was found that PMV did not affect the hospital mortality (OR=1.979, *P*=0.420).

## DISCUSSION

In this trial, we tried to propose predictive independent factors for PMV after cardiac surgery and the effects of these factors on mortality in our heart center. We found that female gender, preoperative level of leukocytosis, intraoperative lactate levels, CPB time and revision for bleeding were independent risk factors for PMV following cardiac surgery with CPB. Despite the current literature, smoking and COPD were not predictive factors for PMV. In our centre, the anesthetists apply effective treatment protocols from the first day of the encounter to hospital until the day of discharge for patients who have asthma or COPD. The successful management could have caused the current results of our study. In addition, PMV did not affect hospital mortality.

Early weaning from mechanical ventilators can provide a shorter stay in the ICU and hospital and facilitate the recovery of cardiopulmonary functions. Despite there is no exact consensus for predictors of PMV in the literature, due to the nature of retrospective study protocols and randomization of patients who underwent various types of cardiac surgery, these studies can provide ability to decrease the intubation time after surgery by identifying perioperative risk factors and demographic data that enhance the tendency for PMV ^[7]^. Rose et al. ^[8]^ revealed a meta-analysis about variations in the definition of prolonged ventilation despite a conference held in 2005 organized by the National Association for Medical Direction of Respiratory Care (NAMDRC) and its delineation for PMV as mechanical ventilation support requiring ≥21 sequential days, ≥6 hours per day, of invasive (by tracheostomy or endotracheal tube) or noninvasive (facial/nasal interface) methods of conveyance; changeable descriptions arise in recent studies. In the results of the analysis of the world literature, the authors mostly accepted the threshold for extubation time of 24 or 48 hours to determine the PMV time ^[10]^. Cohen et al. ^[11]^ evaluated the results of 1,112 patients who underwent CABG and required PMV. The authors used 24 hours as a threshold, similar to our study. They used fast-track extubation protocols at their institution, just as we did to eliminate the actual unstabilized patients much earlier to solve problems in the entire neurological, cardiac, gastrointestinal and pulmonary systems. Kollef et al. ^[12]^ validated that PMV after CABG surgery was related to increased ratio of sepsis, multiorgan dysfunction and mortality. These two articles confirmed that PMV after CABG depends on preoperative renal failure, unstable angina, COPD, prolonged CPB and cross-clamping time and high intraoperative levels of residual fluid balance ^[13]^; jarringly in our report, we found that PMV was independent of aortic cross-clamp (ACX) time, preoperative renal functions and COPD, and besides we incorporated the full blood counts, including platelets, monocytes, lymphocytes, leukocytes, hemoglobin and albumin levels, blood glucose levels and, most important, the intraoperative lactate levels that manifests organ perfusion and oxygenation.

Laine et al. ^[14]^ determined that lactate level ≥4 mmol/L was an independent predictor of major complications including PMV in their report about how isolated high lactate or low central venous oxygen saturation following cardiac surgery was relevant with major complications. As we remarked the importance of the lactate level for indicating the organ perfusion previously, we evaluated the intraoperative lactate levels in this study and the consequences were significant and valuable for predicting PMV.

Ji et al. ^[15]^ reported that preoperative congestive heart failure, hypoalbuminemia, low preoperative PaO_2_ and postoperative anemia were four independent risk factors. Merely, they used 48 hours as a cut-off point and value for extubation time limit and they did not include the non-CABG patients. Jacobs et al. ^[5]^ published an article about variegated ventilation time after CABG surgery and they found that the emergency of the surgery was a significantly valuable predictor for PMV, surprisingly conflicting with previous studies and they used 24 hours as a cut-off value either. Totonchi et al. ^[9]^ reported the predictive factors for delayed extubation such as long CPB time, postoperative revision for bleeding, type of surgery and inotrope dependence were the perioperative predictive factors for delayed extubation. Contrary to previous studies, but similar to our report, they also determined female gender as a predictive factor for PMV. They also found that the timing of surgery does not affect the risk of PMV, unlike our results and the results of previous studies ^[5]^.

Faritous et al. ^[4]^ suggested that age, low EF, pre-existing respiratory or renal failure, reoperation, use of inotropic agents and emergency surgery were the predictors for PMV. In our study, we excluded reoperations to standardize the values. Saleh et al. ^[16]^ reported the study with 10,977 patients who underwent elective coronary artery surgery and PMV occurred in 215 patients due to the cut-off value of 72 hours. They suggested that the predictors for PMV after CABG procedure were age, PVD, HT, NYHA stage, elevated BMI, low FEV1 and prolonged CPB times. Knapik et al. ^[17]^ published an article including reoperations and aortic arch operations either. However, they did not include the hemogram values, lactate levels and existence of postoperative pulmonary infection that were essential ^[10]^.

Previous studies have shown that low EF and hemoglobin levels were predictive factors for PMV either ^[18]^; conversely, we found that there was no significant difference. We found that intraoperative lactate levels, CPB time, female gender and preoperative leukocytosis and postoperative revision for bleeding were significantly different in the PMV group and these are the final predictors in our study.

### Limitations

The retrospective nature of this study can cause some limitations, but in the future this study can develop as a prospective more populated multicenter designated work.

This study provides a new approach to predict PMV after cardiac surgery, including new parameters such as lactate level, a significant indicator for organ perfusion beside oxygenation.

## CONCLUSION

In our report, differently from the previous studies, we revealed that intraoperative lactate levels that manifest organ perfusion and oxygenation were included and were significantly different in the early extubation group than in the PMV group. Among all other predictors, the lactate level is much more valued for indication of postoperative lung dysfunction. Female gender, preoperative leukocytosis, CPB time and lactate levels, and revision for bleeding were the independent predictive factors for PMV. Moreover, PMV did not affect the early-term mortality during hospital stay.

**Table:** 

Authors' roles & responsibilities	
RA	Substantial contributions to the conception or design of the work; or the acquisition, analysis, or interpretation of data for the work; drafting the work or revising it critically for important intellectual content; final approval of the version to be published
AZK	Substantial contributions to the conception or design of the work; or the acquisition, analysis, or interpretation of data for the work; drafting the work or revising it critically for important intellectual content; final approval of the version to be published
DC	Substantial contributions to the conception or design of the work; or the acquisition, analysis, or interpretation of data for the work; drafting the work or revising it critically for important intellectual content; final approval of the version to be published
AE	Substantial contributions to the conception or design of the work; or the acquisition, analysis, or interpretation of data for the work; drafting the work or revising it critically for important intellectual content; final approval of the version to be published
FY	Substantial contributions to the conception or design of the work; or the acquisition, analysis, or interpretation of data for the work; drafting the work or revising it critically for important intellectual content; final approval of the version to be published
ÜY	Substantial contributions to the conception or design of the work; or the acquisition, analysis, or interpretation of data for the work; drafting the work or revising it critically for important intellectual content; final approval of the version to be published
MBR	Substantial contributions to the conception or design of the work; or the acquisition, analysis, or interpretation of data for the work; drafting the work or revising it critically for important intellectual content; final approval of the version to be published
